# Inhibition of adenosine kinase attenuates myocardial ischaemia/reperfusion injury

**DOI:** 10.1111/jcmm.16328

**Published:** 2021-02-01

**Authors:** Wenjun Wang, Bailu Wang, Shukun Sun, Shengchuan Cao, Xiaoxuan Zhai, Chuanxin Zhang, Qun Zhang, Qiuhuan Yuan, Yi Sun, Mengyang Xue, Jingjing Ma, Feng Xu, Shujian Wei, Yuguo Chen

**Affiliations:** ^1^ Department of Emergency and Chest Pain Center Qilu Hospital Cheeloo College of Medicine Shandong University Jinan China; ^2^ Clinical Research Center for Emergency and Critical Care Medicine of Shandong Province Institute of Emergency and Critical Care Medicine of Shandong University Qilu Hospital Cheeloo College of Medicine Shandong University Jinan China; ^3^ Key Laboratory of Emergency and Critical Care Medicine of Shandong Province Key Laboratory of Cardiopulmonary‐Cerebral Resuscitation Research of Shandong Province Qilu Hospital Cheeloo College of Medicine Shandong University Jinan China; ^4^ The Key Laboratory of Cardiovascular Remodeling and Function Research Chinese Ministry of Education Chinese Ministry of Health and Chinese Academy of Medical Sciences, Qilu Hospital, Cheeloo College of Medicine, Shandong University Jinan China; ^5^ The State and Shandong Province Joint Key Laboratory of Translational Cardiovascular Medicine Qilu Hospital Cheeloo College of Medicine Shandong University Jinan China; ^6^ Clinical Trial Center, Qilu Hospital Cheeloo College of Medicine Shandong University Jinan China

**Keywords:** adenosine kinase, apoptosis, myocardial ischaemia/reperfusion injury, necroptosis, X‐linked inhibitor of apoptosis protein

## Abstract

Increased adenosine helps limit infarct size in ischaemia/reperfusion‐injured hearts. In cardiomyocytes, 90% of adenosine is catalysed by adenosine kinase (ADK) and ADK inhibition leads to higher concentrations of both intracellular adenosine and extracellular adenosine. However, the role of ADK inhibition in myocardial ischaemia/reperfusion (I/R) injury remains less obvious. We explored the role of ADK inhibition in myocardial I/R injury using mouse left anterior ligation model. To inhibit ADK, the inhibitor ABT‐702 was intraperitoneally injected or AAV9 (adeno‐associated virus)—ADK—shRNA was introduced via tail vein injection. H9c2 cells were exposed to hypoxia/reoxygenation (H/R) to elucidate the underlying mechanisms. ADK was transiently increased after myocardial I/R injury. Pharmacological or genetic ADK inhibition reduced infarct size, improved cardiac function and prevented cell apoptosis and necroptosis in I/R‐injured mouse hearts. In vitro, ADK inhibition also prevented cell apoptosis and cell necroptosis in H/R‐treated H9c2 cells. Cleaved caspase‐9, cleaved caspase‐8, cleaved caspase‐3, MLKL and the phosphorylation of MLKL and CaMKII were decreased by ADK inhibition in reperfusion‐injured cardiomyocytes. X‐linked inhibitor of apoptosis protein (XIAP), which is phosphorylated and stabilized via the adenosine receptors A2B and A1/Akt pathways, should play a central role in the effects of ADK inhibition on cell apoptosis and necroptosis. These data suggest that ADK plays an important role in myocardial I/R injury by regulating cell apoptosis and necroptosis.

## 1 INTRODUCTION

Early reperfusion using thrombolytic therapy or primary percutaneous coronary intervention is the most effective therapeutic intervention for limiting myocardial infarct size.^1^, ^2^, ^3^ However, the acute restoration of blood flow within the epicardial coronary artery leads to the generation of oxidative stress, calcium overload and inflammation, which induces further cardiomyocyte death, a phenomenon known as myocardial ischaemia/reperfusion (I/R) injury.^2^ I/R‐induced cell death of cardiomyocytes may account for up to 50% of the final myocardial infarct size.^1^ Although many modes of cell death including necrosis, apoptosis, necroptosis and autophagy contribute to the loss of cardiomyocytes during I/R injury^4^ and their quantitative contribution to final infarct size is less obvious, pharmacological or genetic inhibition of cell death reduces infarct size.^5^, ^6^

Adenosine is a purine nucleotide that mediates a wide variety of physiological effects including inflammation, immune reaction, cell proliferation, angiogenesis and metabolism.^7^, ^8^ Adenosine is generated both inside and outside the cell through the breakdown of adenosine triphosphate (ATP).^9^ Intracellular adenosine can also be produced by the hydrolysis of *S*‐adenosylhomocysteine (SAH), which is converted by SAH hydrolase.^10^ Extracellular adenosine functions as a signalling molecule that activates four known adenosine receptors, A_1_, A_2A_, A_2B_ and A_3_.^8^ Increased levels of adenosine also elevate the content of SAH, which is a potent feedback inhibitor of methyltransferases and thus constrains transmethylation reactions.^9^ Although the effects of adenosine on cell apoptosis are inconsistent in different cells, it plays an important role in preventing reperfusion‐induced cardiomyocyte apoptosis and limiting myocardial infarct size.^11^, ^12^ Moreover, intravenous adenosine reduced infarct size in ST‐segment elevation myocardial infarction patients undergoing reperfusion therapy.^13^, ^14^ However, clinical outcome including mortality and congestive heart failure in patients reperfused beyond 3 hours was not improved by adenosine.^15^

Extracellular adenosine can be transported into the cell via concentrative or equilibrative nucleoside transporters.^16^ Up to 70% of myocardial interstitial adenosine is transported into cardiomyocytes.^17^ Intracellular adenosine is converted to 5‐adenosine monophosphate via adenosine kinase (ADK) or to inosine via adenosine deaminase (ADA).^18^ ADK has a high affinity with a lower Michaelis constant (1‐2 mmol/L) for adenosine and is regarded as the principal enzyme in regulating intracellular adenosine levels.^9^ Up to 90% of adenosine in cardiomyocytes is converted to AMP by ADK.^19^ Recently, the critical role of ADK in several pathologies including diabetes, epilepsy and cancer has attracted attention.^9^ ADK inhibition protects against renal I/R injury by limiting oxidative stress, inflammation and cell apoptosis, but the role of ADK in myocardial I/R injury has never been investigated.^20^

In the present study on the effects of pharmacological or genetic inhibition of ADK on myocardial I/R injury in vivo and in vitro, ADK inhibition decreased reperfusion‐induced cell apoptosis and necroptosis via the A_2B_ adenosine receptor/Akt/XIAP pathway. Targeting ADK may be a potential therapeutic target for myocardial I/R injury.

## 2 METHODS

### 2.1 Reagents and antibodies

ABT‐702 was from Merck Millipore (116890, Shanghai). MK‐2206 was from Selleck (S7025/S1078, Shanghai, 10 μmol/L). 8(p‐Sulfophenyl)theophylline (8‐SPT) was from Absin (abs42012139, Shanghai, 100 μmol/L). DPCPX was from Abcam (ab120396, Shanghai, 100 nmol/L). MRS 1754 was from MedChemExpress (HY‐14121, Shanghai, 10 nmol/L). Primary antibodies against the following targets were obtained from the sources listed: ADK (Santa Cruz, Heidelberg, 514588), XIAP (CST, 2042), phosphor‐XIAP antibody (p‐SER87) (CST, 193315), RIP1 (CST, 3493), phosphor‐RIP1 antibody (Ser166)(CST, 31122), RIP3 (CST, 15828), phosphor‐RIP3 (Abcam, ab195117), caspase‐3 (CST, 9662), caspase‐8 (CST, 4790), caspase‐9 (CST, 9504), caspase‐12 (CST, 2202), Bcl2 (CST, 3498), Bax (CST, 2772), β‐actin (Proteintech, 6008‐1‐Ig), Akt (CST, 4685), phosphor‐Akt (Ser473) (CST, 4060), P38 (CST, 8690), phosphor‐P38 (CST, 4511), MLKL (Proteintech, 66675), phosphor‐MLKL (Abcam, ab196436). CaMKII (CST, 3362), phosphor‐CaMKII (Abcam, ab32678) and adenosine receptor A_2B_ (GeneTex, GTX132217). Secondary antibodies were from Cell Signaling Technology. Primary antibodies were diluted at 1:1000, and secondary antibodies were diluted at 1:5000.

### 2.2 Animal myocardial ischaemia/reperfusion injury models

C57BL/6 mice were maintained on a 12‐hour dark/light cycle with unlimited access to water and chow. Male mice aged 8‐12 weeks were used to establish an acute myocardial ischaemia/reperfusion injury model by ligation of the left anterior descending artery (LAD). Briefly, mice were anaesthetized with isoflurane inhalation, intubated with an intravenous catheter and ventilated with a Rodent Anesthesia Machine. For ischaemia, the LAD was ligated with 6‐0 silk sutures threaded through a snare. The occlusion was maintained for 30 minutes, and then, the snare was released to achieve reperfusion for 4 or 24 hours. The sham group underwent the same procedures without ligating the LAD. ABT702 (2 mg/kg) or vehicle was intraperitoneally injected 30 minutes before I/R surgery. All aspects of mouse care and experimentation were performed in accordance with the Guide for the Care and Use of Laboratory Animals and approved by the Institutional Animal Care and Use Committee of Shandong University.

### 2.3 Infarct size measurement

After reperfusion for 4 hours, mice were killed for tissue harvesting. To measure the myocardial infarct size, the hearts were perfused with 1% Evans blue dye to indicate the area at risk after occluding the LAD at the same place and then sliced into 2‐mm‐thick slices. Then, the slices were incubated in 1% 2,3,5‐triphenyltetrazolium chloride (TTC) solution at 37°C for 20 minutes. Images were photographed and analysed using the ImageJ software (NIH).

### 2.4 Cardiac function

At the end of the 24‐hour reperfusion, transthoracic echocardiography was performed in mice under anaesthesia using isoflurane inhalation (VEVO 2100, VisualSonics). Left ventricular ejection fraction (LVEF) and fractional shortening (LVFS) were recorded to evaluate cardiac function.

### 2.5 Serum lactate dehydrogenase (LDH) assay and myocardial S‐Adenosylhomocysteine (SAH) test

Blood was centrifuged at 2500 *g*, 4°C for 15 minutes, and then, serum was collected for determination of LDH using an LDH Assay Kit (ab102526, Abcam). Myocardial SAH levels were examined with an ELISA kit (Cell Biolabs, STA‐671).

### 2.6 Transmission electron microscopy

Transmission electron microscopy was performed to visualize the morphology of mitochondria. After fixing overnight in 2.5% glutaraldehyde, ultrathin heart sections of 80 nm thickness were rinsed in 0.1 mmol/L cacodylate buffer with 1% tannic acid and postfixed in 1% osmium tetroxide in 0.1 mmol/L cacodylate buffer for 1 hour. Then, the samples were rinsed again and dehydrated with alcohol and embedded in Epon 812. Finally, the sections were observed using a transmission electron microscope (FEI Talos F200C, 200 kV).

### 2.7 Cell culture

H9c2 cells (ATCC, rat) were cultured in DMEM (Gibco) supplemented with 10% foetal bovine serum (FBS, HyClone) and maintained in a humidified incubator with 95% air/5%

CO_2_ at 37°C. Neonatal rat cardiomyocytes were isolated from 2 to 4 day‐old Sprague Dawley rats by enzymatic digestion and cultured in serum‐containing DMEM.^21^ For hypoxia/reoxygenation, the cells were subjected to low‐glucose serum‐free DMEM and placed in a hypoxia chamber (1% O_2_) for 12 hours. Following hypoxia, the cells were switched to the regular culture medium and placed in a normoxic incubator for 1‐4 hours.

### 2.8 Apoptosis assay

To assess cell apoptosis in I/R‐injured heart tissues or cardiomyocytes, TUNEL (Roche) was performed according to the manufacturer's instructions. The apoptotic index was calculated as TUNEL‐positive cells/total cells.

### 2.9 Necroptosis assay

Cell necroptosis in I/R‐injured hearts was detected using double staining with Evans blue dye (EBD) and CaV3 antibody.^22^ Mice were intraperitoneally injected with EBD (10 mg/mL) 14 hours before I/R injury. After the operation, the hearts were separated and frozen in liquid nitrogen. Cryosections of hearts were then immunostained with CaV3 antibody. Sections were observed and imaged using a fluorescence microscope (Olympus) and analysed using the ImageJ software (NIH). Necroptotic cells after hypoxia/reoxygenation treatment were detected by flow cytometry with PI/Annexin V Apoptosis Detection kits and analysed using CytExpert software (Beckman Coulter).

### 2.10 ADK knockdown by AAV9 virus vectors

AAV9‐ADK‐shRNA viruses were constructed by GeneChem and were intravenously administered via the tail vein 3 weeks before I/R surgery. For control group, AAV9‐scramble shRNA was introduced.

### 2.11 X‐linked inhibitor of apoptosis protein (XIAP) knockdown by siRNA

XIAP was knocked down in H9c2 cells using siRNA. The interfering efficiency of 3 pairs of siRNAs was tested (Table S1).

### 2.12 Western blot

Lysed proteins from cardiac tissue or H9c2 cells were separated by SDS‐PAGE and electrotransferred to PVDF membranes (Millipore). After incubation with primary antibodies and subsequent corresponding horseradish peroxidase‐conjugated secondary antibodies, blots were visualized with chemiluminescence reagents (Invitrogen) and analysed using the ImageJ software.

### 2.13 Real‐time quantitative PCR

mRNA expression of adenosine receptors in myocardium tissues and cells was evaluated by real‐time quantitative PCR. An EASYspin plus RNA extraction kit (Aidlab, China) was used to extract RNA according to the instructions. PrimeScript RT Master Mix (Takara) was then used to reverse‐transcribe RNA to DNA. The amplifications and measurements were performed on an ABI 7500 quantitative polymerase chain reaction instrument (Applied Biosystems, Thermo Fisher Scientific). 2^−ΔΔ^ CT of data from 6 independent experiments were recorded and analysed. The primer sequences are shown in Table S2.

### 2.14 Detection of mitochondrial superoxide production

Mitochondrial superoxide was assessed using MitoSox™ Red (Invitrogen). Cells were observed with a fluorescence microscope.

### 2.15 Mitochondrial membrane potential (ΔΨm)

ΔΨm was detected using the JC‐1 (Beyotime) method. Briefly, cells were incubated with JC‐1 in serum‐free medium for 20 minutes at 37°C. After washing, cells were observed and imaged under a fluorescence microscope (Olympus). ΔΨm was calculated using the red/green fluorescence ratio.

### 2.16 Mitochondrial permeability transition pore (mPTP)

Opening of the mPTP was monitored by loading cells with calcein‐AM (Sigma‐Aldrich) and CoCl_2_.^23^ H9c2 cells were first incubated with calcein‐AM (2 μmol/L) and CoCl_2_ (1 mmol/L) for 35 minutes at room temperature and then washed with CoCl_2_. Basic fluorescence was measured at ex/em wavelengths of 488/515 nm. Subsequently, cells were treated with H_2_O_2_ (100 μmol/L) for 120 minutes at 37°C and fluorescence was measured at different time‐points. Opening of the mPTP was indicated by the abrupt loss of fluorescence and expressed as a percentage of the baseline fluorescence intensity.

### 2.17 ATP test

ATP levels were determined using an ATP Assay Kit (Beyotime) and were normalized to total protein content, which was evaluated by the bicinchoninic acid method.

### 2.18 Statistical analysis

The data were presented as the means ± SEM after passing normality or equal variance tests. Student's *t* test or one‐way analysis of variance followed by Tukey's post hoc test was used to compare statistical significance. Statistical significance was considered at *P* < .05. All data were analysed using GraphPad Prism version 6.0.

## 3 RESULTS

### 3.1 ADK inhibition reduces myocardial injury and improves cardiac function after I/R

**FIGURE 1:**
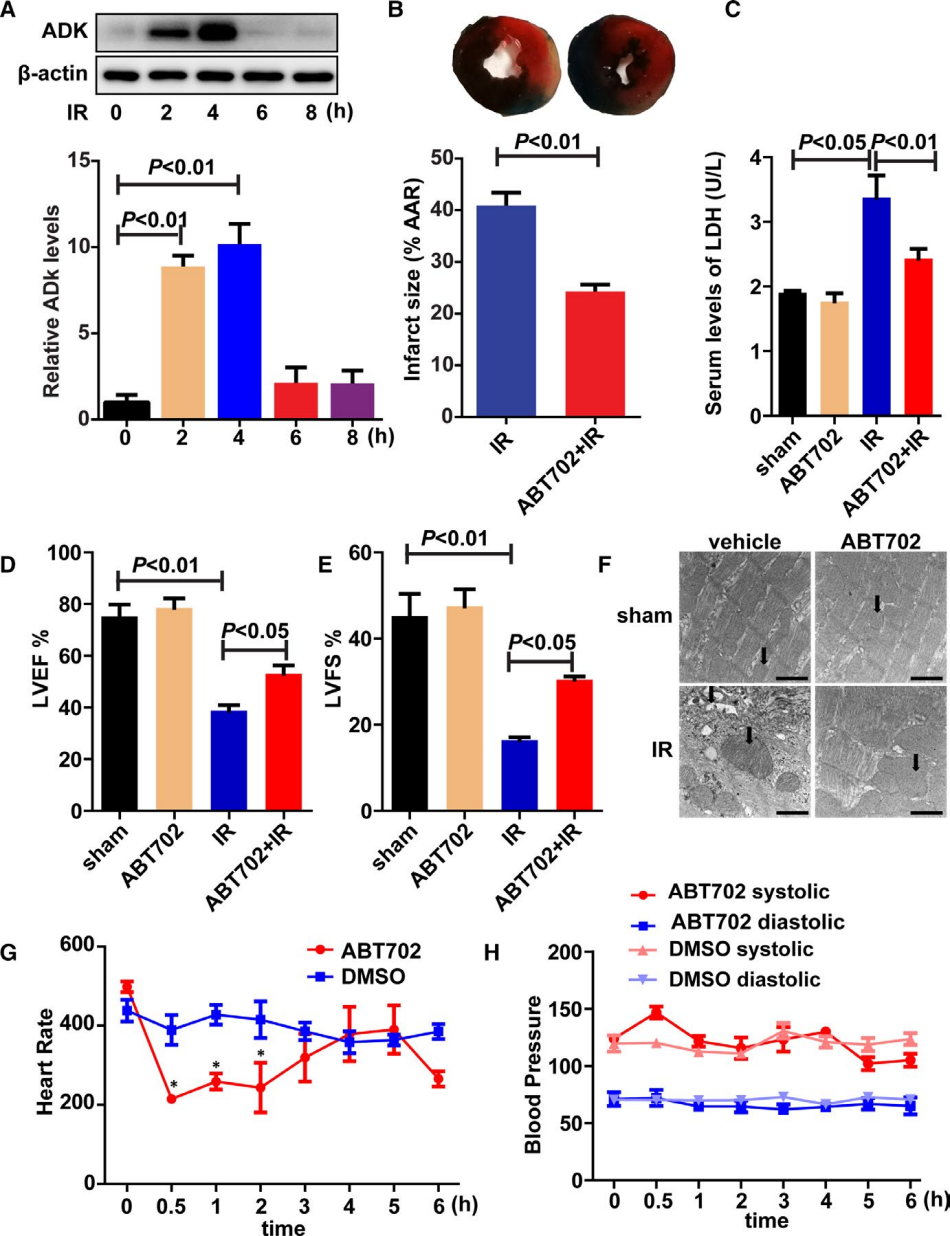
ADK inhibition prevents myocardial I/R injury and improves cardiac function. A, The expression of ADK from I/R‐injured hearts was immunoblotted and quantitatively analysed. (n = 5). B, Infarct size was visualized by Evans blue/TTC staining and was quantitatively analysed. (n = 10). C, LDH was determined in the serum from mice exposed to myocardial 30‐min ischaemia/2‐h reperfusion. (n = 5). D and E, Cardiac function was evaluated via echocardiography, as indicated by LVEF and LVFS at the end of the 24‐h reperfusion. (n = 8). F, Myocardial ultrastructure changes were observed by electron microscope. (n = 5). Scale bar = 0.5 μm. G and H, Mouse heart rates and blood pressures were measured by tail‐cuff plethysmography (MRBP, IITC Life Science). (n = 6). *P < .05 vs DMSO group

The expression of ADK was transiently increased from 2 to 4 hours and returned to normal levels after 6 hours of reperfusion (Figure 1A). The ADK inhibitor ABT702 significantly reduced myocardial infarct size compared with that of the I/R group (Figure 1B). To confirm the effect of ADK on myocardial infarct size after I/R injury, the expression of myocardial ADK was knocked down using AAV9 vectors (Figure S1A). Compared with the blank vector group, knockdown of ADK significantly limited the enlargement of myocardial infarct size after I/R injury (Figure S1B,C). LDH release was also reduced by ADK inhibition (Figure 1C). To evaluate the effect of ADK inhibition on cardiac function after I/R injury, mice were subjected to 30‐minute LAD ligation and 24‐hour reperfusion. Both LVEF and LVFS were improved by pre‐treatment with ADK inhibitor or ADK knockdown (Figure 1D,E and Figure S1D,E). Furthermore, we observed the morphology of the myocardium from different groups using an electron microscope. Clear muscle segments and normal mitochondria were found in the sham group, while damaged muscle segments and swollen mitochondria were observed in the I/R group (Figure 1F). However, ADK inhibition markedly reduced I/R‐induced damage to muscle segments and mitochondria (Figure 1F). Moreover, ADK inhibition reduced the heart rate but did not affect blood pressures in mice (Figure 1G,H).

### 3.2 ADK inhibition prevents I/R‐induced cell apoptosis and necroptosis

**FIGURE 2:**
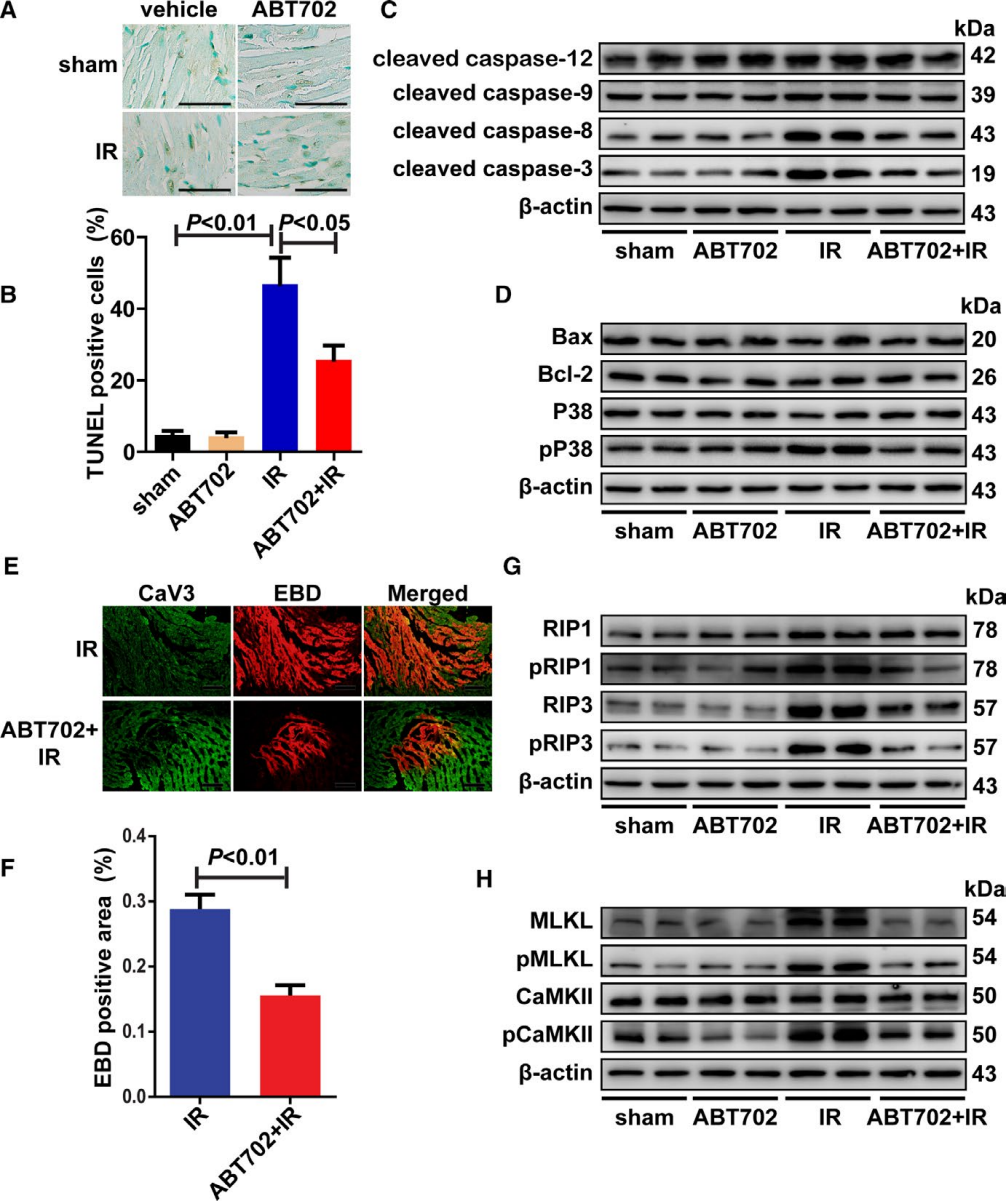
Cell apoptosis and necroptosis are decreased by ADK inhibition in I/R‐injured hearts. A and B, Apoptotic cells in the area at risk were determined by TUNEL staining and quantitatively analysed. (n = 5). Scale bar = 20 μm. C and D, Protein lysates from I/R‐injured hearts (30‐min ischaemia/4‐h reperfusion) were immunoblotted for cleaved caspase‐12, cleaved caspase‐9, cleaved caspase‐8, cleaved caspase‐3, Bax, Bcl‐2, phosphor‐P38 and P38. (n = 5). E and F, Myocardial necrosis were evaluated by double staining with CaV3 (viable cardiomyocytes) and EBD (necrotic cells). (n = 5). Scale bar = 50 μm. G and H, Protein lysates from I/R‐injured hearts (30‐min ischaemia/4‐h reperfusion) were immunoblotted for RIP1, phosphor‐RIP1, RIP3, phosphor‐RIP3, MLKL, phosphor‐MLKL, CaMKII and phosphor‐CaMKII (n = 5)

TUNEL staining revealed that ADK inhibition decreased the apoptotic cells in the area at risk compared with that in the I/R group (Figure 2A,B). The initiation step of cell apoptosis can be divided into extrinsic and intrinsic pathways in which caspase‐8 and caspase‐9 play critical roles.^24^ Caspase‐12 mediates the initiation of endoplasmic reticulum stress‐induced cell apoptosis.^25^ In this study, reperfusion increased the activation of caspase‐12, caspase‐9, caspase‐8 and caspase‐3 in heart tissues and ADK inhibition suppressed the increase in the activation of caspase‐9, caspase‐8 and caspase‐3 but not the increases in the activation of caspase‐12 in I/R‐injured hearts (Figure 2C and Figure S2B). Bax and Bcl‐2 are important mediators of the transduction of death signals to mitochondria.^26^ However, we found that the contents of Bax and Bcl‐2 were unchanged by I/R injury or by ADK inhibition in heart tissues (Figure 2D and Figure S2C). Consistent with previous studies, P38 mitogen‐activated protein kinase (P38 MAPK) was activated by I/R‐induced cellular stress (Figure 2D and Figure S2C).^27^ Interestingly, ADK inhibition suppressed the phosphorylation of P38 MAPK in I/R‐injured hearts (Figure 2D and Figure S2C). Necroptosis, a new form of programmed necrosis, also exerts an important effect on myocardial I/R injury.^22^, ^28^ ADK inhibition decreased I/R‐induced myocardial necrosis, as indicated by CaV3 and EBD staining (Figure 2E,F). Receptor‐interacting serine/threonine‐protein kinase (RIP1) and the phosphorylation of RIP1 were both increased by I/R injury but were not changed by ADK inhibition (Figure 2G and Figure S2D). I/R injury increased the content of RIP3 and the phosphorylation of RIP3, whereas ADK inhibition prevented the I/R‐induced increase in RIP3 and the phosphorylation of RIP3 (Figure 2G and Figure S2D). The expression of mixed lineage kinase domain‐like pseudokinase (MLKL), the phosphorylation of MLKL and the phosphorylation of calcium‐calmodulin‐dependent protein kinase II (CaMKII) were increased after I/R injury, which was prevented by ADK inhibition (Figure 2H and Figure S2E). Moreover, neither I/R injury nor ADK inhibition affected the content of CaMKII in heart tissues (Figure 2H and Figure S2E).

### 3.3 ADK inhibition reduces cell apoptosis and necroptosis in H/R‐injured H9c2 cells

**FIGURE 3:**
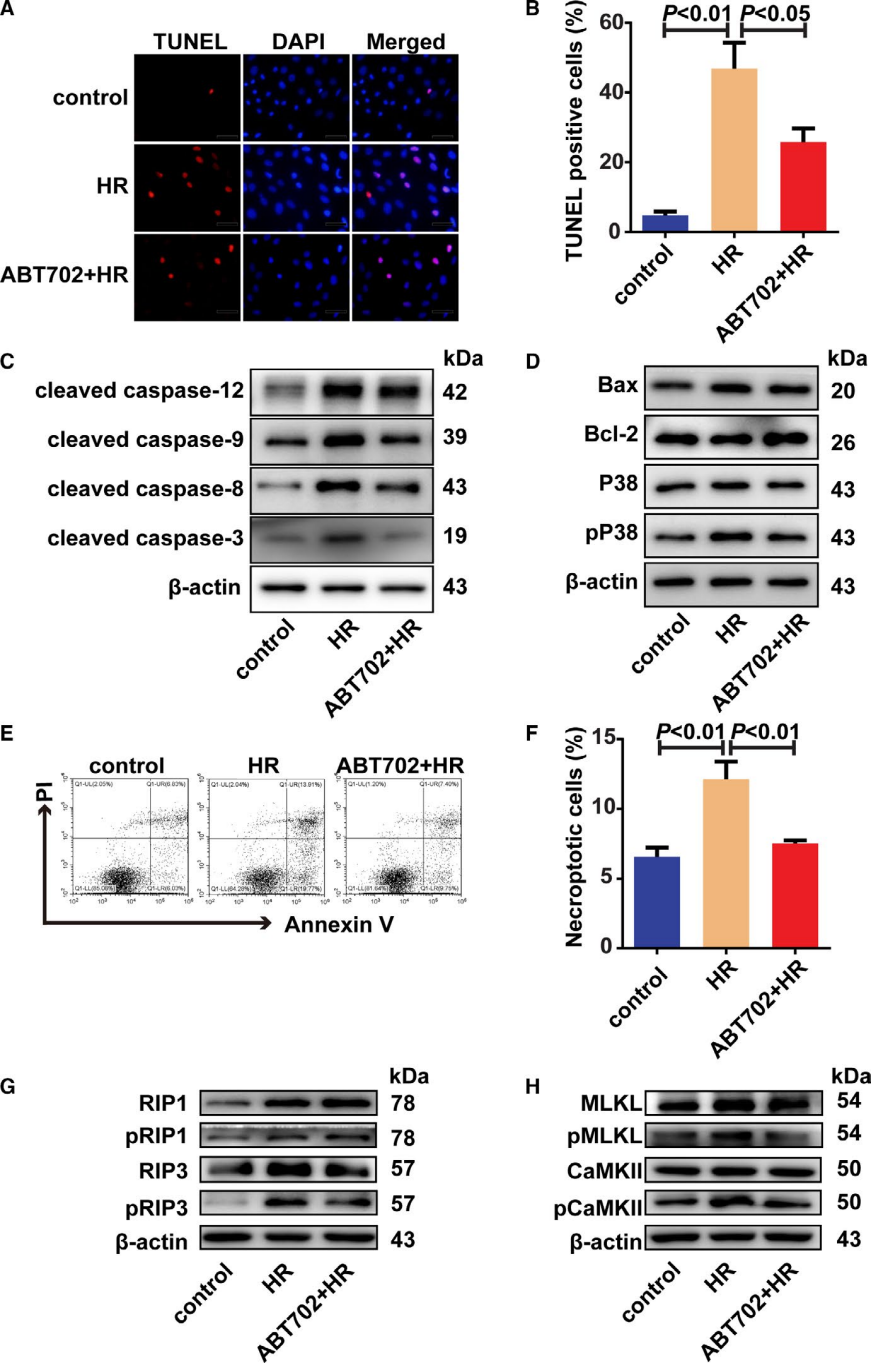
Cell apoptosis and necroptosis are decreased by ADK inhibition in vitro. H9c2 cells were incubated in hypoxia chamber for 12 h and exposed to reoxygenation for 4 h with/without ABT‐702 (1 μmol/L) pre‐treatment. A and B, Apoptotic cells were stained by TUNEL method and quantitatively analysed. (n = 5). Scale bar = 20 μm. C and D, Representative immunoblots of cleaved caspase‐12, cleaved caspase‐9, cleaved caspase‐8, cleaved caspase‐3, Bax, Bcl‐2, phosphor‐P38 and P38 in H/R‐stimulated H9c2 cells. (n = 5). E and F, Necroptotic cells were analysed by Annexin V/PI staining using flow cytometry. (n = 5). G and H, Representative immunoblots of RIP1, phosphor‐RIP1, RIP3, phosphor‐RIP3, MLKL, phosphor‐MLKL, CaMKII and phosphor‐CaMKII in H/R‐stimulated H9c2 cells (n = 5)

To further verify the effects of ADK inhibition on cell apoptosis and cell necroptosis, H9c2 cells were cultured and subjected to H/R injury. Apoptotic cells were significantly reduced by ADK inhibition in H/R‐injured H9c2 cells (Figure 3A,B). H/R stimulation increased the levels of cleaved caspase‐12, caspase‐9, caspase‐8 and caspase‐3 (Figure 3C and Figure S3A). Similar to the in vivo results, ADK inhibition only suppressed the activation of caspase‐9, caspase‐8 and caspase‐3 in H/R‐treated H9c2 cells (Figure 3C and Figure S3A). Additionally, ADK inhibition suppressed the phosphorylation of P38 MAPK but had no effect on the content of Bax, Bcl‐2 and P38 MAPK (Figure 3D and Figure S3B). Necroptotic cells were identified by cell flow cytometry as Annexin V and PI double‐positive cells. H/R promoted cell necroptosis, and ADK inhibition decreased H/R‐induced necroptotic cells (Figure 3E,F). H/R stimulation increased RIP1, the phosphorylation of RIP1, RIP3 and the phosphorylation of RIP3; however, only RIP3 and the phosphorylation of RIP3 were effectively decreased by ADK inhibition (Figure 3G and Figure S3C). Further study indicated that ADK inhibition prevented H/R‐induced increase in MLKL, and the phosphorylation of MLKL and CaMKII (Figure 3H and Figure S3D).

### 3.4 XIAP mediates the protective role of ADK inhibition in cell apoptosis and necroptosis

**FIGURE 4:**
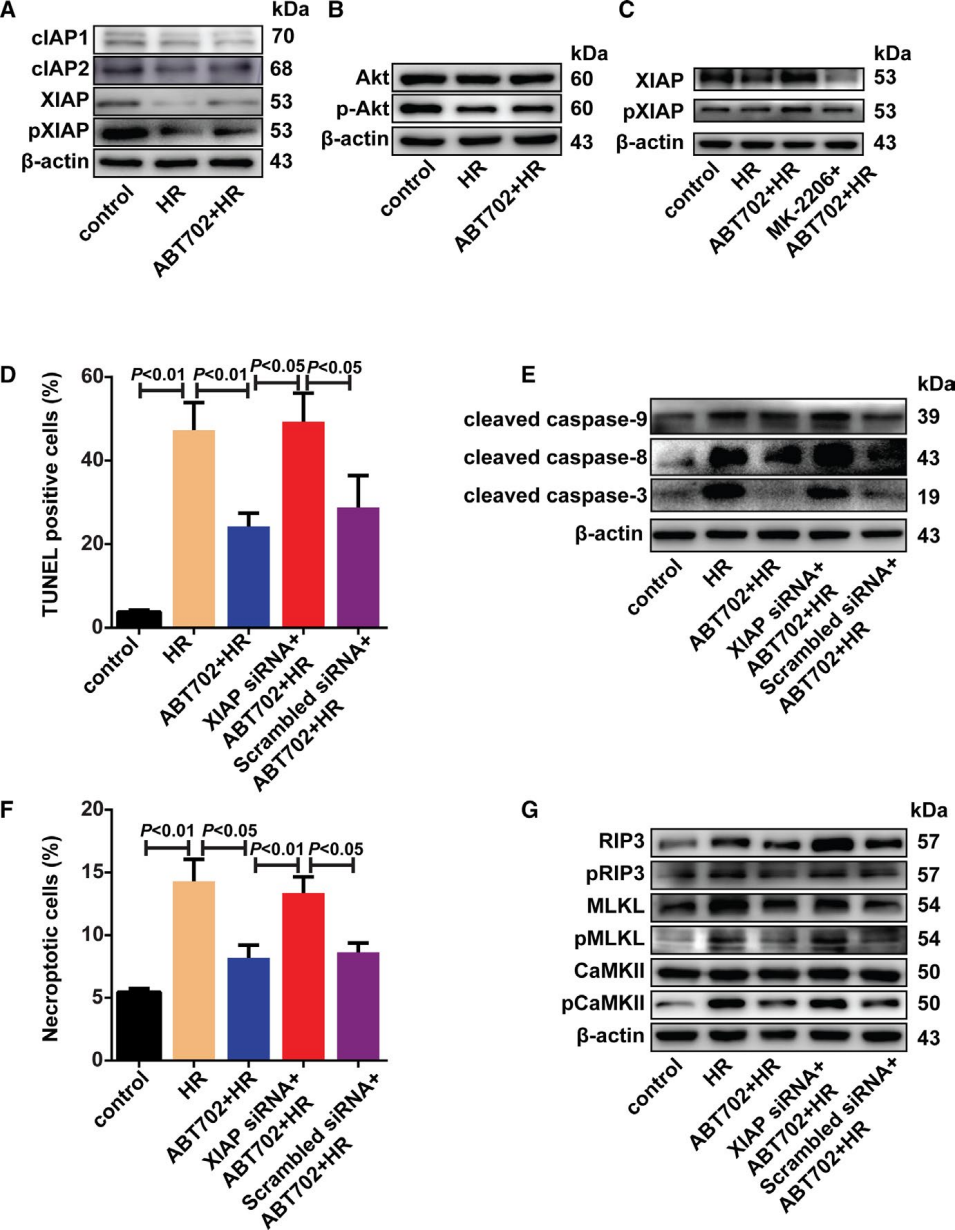
ADK inhibition prevents H/R‐induced cell apoptosis and necroptosis via Akt/XIAP pathway. A, H9c2 cells were incubated in hypoxia chamber for 12 h and exposed to reoxygenation for 4 h. cIAP1, cIAP2, XIAP and phosphor‐XIAP were immunoblotted in H/R‐stimulated H9c2 cells with/without ABT‐702 pre‐treatment. (n = 5). B, H9c2 cells were incubated in hypoxia chamber for 12 h and exposed to reoxygenation for 1 h with/without ADK pre‐treatment. Lysates were separated and immunoblotted for Akt and phosphor‐Akt. (n = 5). C, H9c2 cells were pre‐treated with MK‐2206 (10 μmol/L) before H/R and ADK inhibition. Lysates were immunoblotted for XIAP and phosphor‐XIAP. (n = 5). D, H9c2 cells were transfected with XIAP‐siRNA or scrambled siRNA. Apoptotic cells were stained by TUNEL method and quantitatively analysed. (n = 6). E, Cleaved caspase‐9, cleaved caspase‐8 and cleaved caspase‐3 were immunoblotted. (n = 5). F, Necroptotic cells were analysed by Annexin V/PI staining using flow cytometry. (n = 6). G, RIP3, phosphor‐RIP3, MLKL, phosphor‐MLKL, CaMKII and phosphor‐CaMKII were immunoblotted. (n = 5)

The inhibitors of apoptosis proteins (IAPs), notably cellular IAP1 (cIAP1), cIAP2 and X chromosome–linked IAP (XIAP), are critical and universal regulators of the cell death signalling pathway.^29^, ^30^ Neither cIAP1 nor cIAP2 levels were changed by H/R treatment with/without ADK inhibition (Figure 4A and Figure S4A). However, less XIAP and phosphor‐XIAP were detected by immunoblotting after H/R stimulation compared with control cells; ADK inhibition reversed these changes (Figure 4A and Figure S4A). Because phosphorylation of XIAP at Ser87 by Akt prevents XIAP autoubiquitylation and stabilizes the protein, we tested the effect of ADK inhibition on Akt and its phosphorylation at Ser473.^31^ H/R injury decreased the phosphorylation of Akt at Ser473, which was restored by ADK inhibition (Figure 4B and Figure S4A). Using the Akt inhibitor MK‐2206 abrogated the effect of ADK inhibition on XIAP and phosphorylation of XIAP (Figure 4C and Figure S4B). These findings demonstrated that Akt activation mediates the effect of ADK inhibition on XIAP and phosphorylation of XIAP. To further evaluate the role of XIAP in ADK‐mediated cell death, we then knocked down XIAP using small interfering RNA (siRNA) (Figure S4C). XIAP silencing prevented the protective effect of ADK inhibition on H/R‐induced cell apoptosis and activation of caspase‐9, caspase‐8 and caspase‐3 (Figure 4D,E and Figure S4D). XIAP silencing also eliminated the effect of ADK inhibition on H/R‐induced cell necroptosis (Figure 4F). Moreover, the changes in RIP3, MLKL and the phosphorylation of RIP3 and CaMKII caused by ADK inhibition were also prevented by XIAP knockdown (Figure 4G and Figure S4E).

### 3.5 Adenosine receptors contribute to the protective role of ADK inhibition in H/R‐induced cell death

**FIGURE 5:**
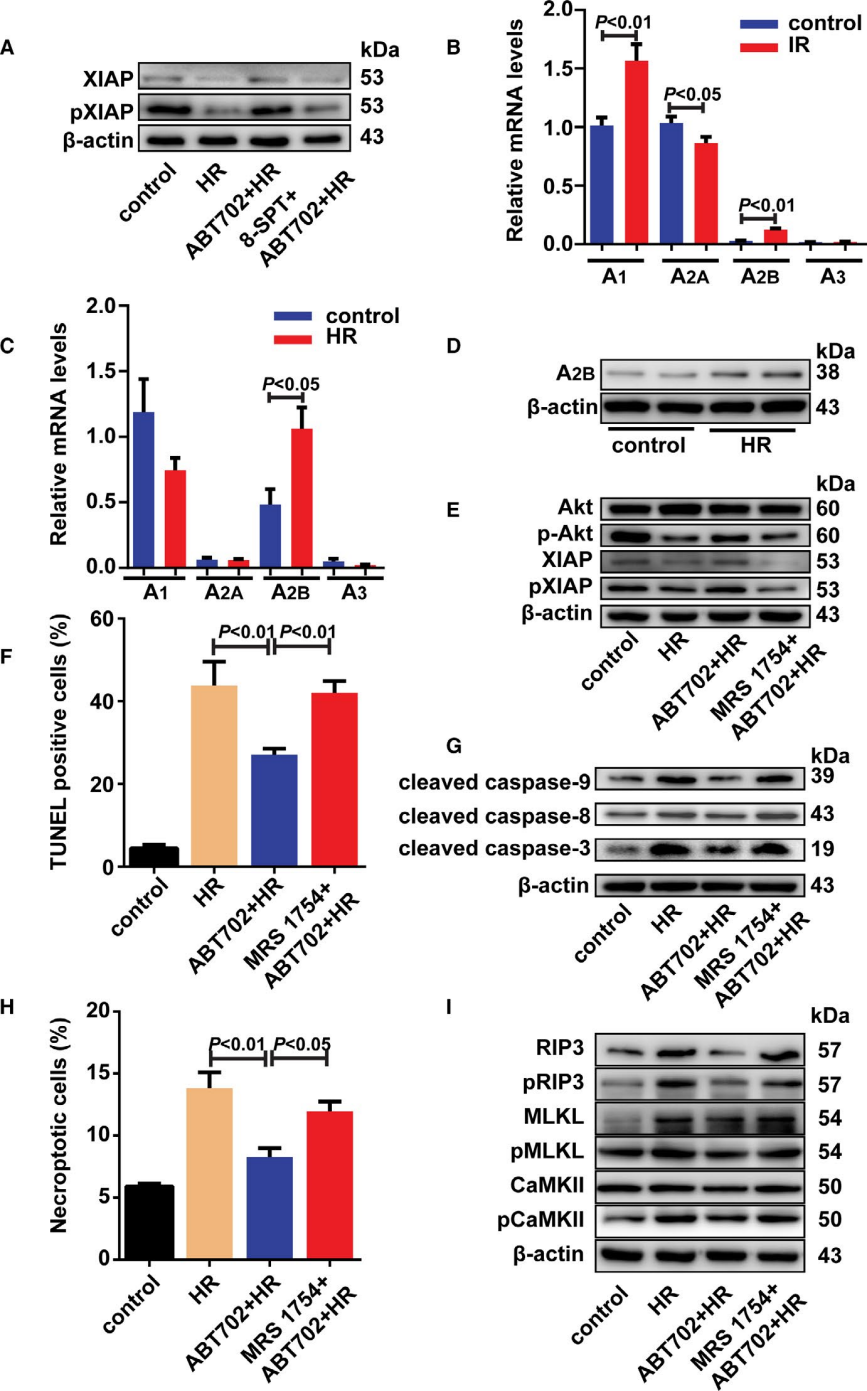
Adenosine receptor A2b mediated the protective effect of ADK inhibition on H/R‐induced cell death. A, H9c2 cells were exposed to H/R injury and pre‐treated with ABT‐702 and/or 8‐SPT (100 μmol/L). Lysates were immunoblotted for XIAP and phosphor‐XIAP. (n = 5). B and C, mRNA expression of adenosine receptors in I/R‐injured mouse hearts, H/R‐treated H9c2 cells and their control were quantitatively analysed. (n = 6). D, Adenosine receptor A_2B_ was immunoblotted in H/R‐treated H9c2 cells. (n = 5). E, H9c2 cells were exposed to H/R injury and pre‐treated with ABT‐702 and/or MRS 1754 (10 nmol/L). Lysates were immunoblotted for Akt, phosphor‐Akt, XIAP and phosphor‐XIAP. (n = 5). F, Apoptotic cells were stained by TUNEL method and quantitatively analysed. (n = 6). G, Cleaved caspase‐9, cleaved caspase‐8 and cleaved caspase‐3 were immunoblotted. (n = 5). H, Necroptotic cells were analysed by Annexin V/PI staining using flow cytometry. (n = 6). I, RIP3, phosphor‐RIP3, MLKL, phosphor‐MLKL, CaMKII and phosphor‐CaMKII were immunoblotted. (n = 5)

ADK inhibition results in increased extracellular adenosine, which leads to the activation of adenosine receptors.^32^, ^33^ Using an adenosine receptor antagonist, the increase in XIAP and the phosphorylation of XIAP induced by ADK inhibition were attenuated in H/R‐treated H9c2 cells (Figure 5A and Figure S5A). To determine which adenosine receptor is critical for the effects of ADK inhibition on I/R‐injured hearts, the mRNA expression of adenosine receptors was analysed in mouse hearts and H9c2 cells. Although the A_1_ receptor was abundantly expressed in mouse hearts and H9c2 cells, the A_1_ receptor was only increased by I/R injury in mouse hearts but not in H9c2 cells (Figure 5B,C). Interestingly, the A_2B_ receptor was significantly increased in both I/R‐injured mouse hearts and H/R‐injured H9c2 cells (Figure 5B,C). Moreover, the protein level of A_2B_ was also increased in H/R‐treated H9c2 cells (Figure 5D). Blockade of the A_2B_ receptor using an A_2B_ receptor antagonist effectively prevented the effects of ADK inhibition on XIAP and the phosphorylation of Akt and XIAP (Figure 5E and Figure S5B). Blockade of the A_2B_ receptor destroyed the protective effects of ADK inhibition on H/R‐induced cell apoptosis and necroptosis (Figure 5F,H). Moreover, changes induced by ADK inhibition in caspase‐9, caspase‐8, caspase‐3, RIP3, MLKL and the phosphorylation of RIP3, MLKL and CaMKII were reversed by blockade of the A_2B_ receptor (Figure 5G,I and Figure S5C,D). Using neonatal rat cardiomyocytes, the critical role of the A_2B_ receptor in the regulation of H/R injury by ADK inhibition in cardiomyocytes was further confirmed (Figure S6A‐C).

### 3.6 ADK inhibition improves mitochondrial function and prevents the production of ROS

**FIGURE 6:**
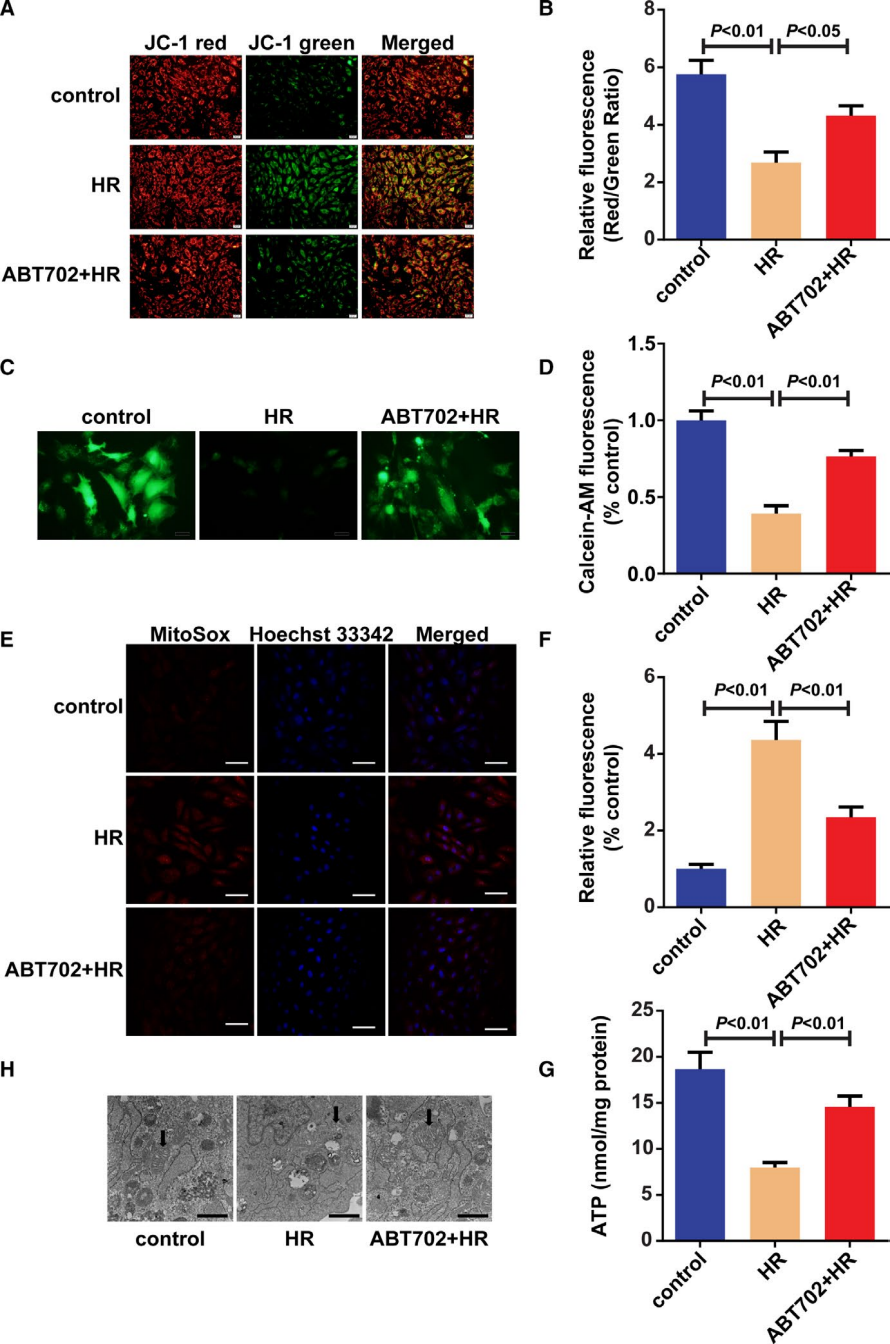
ADK inhibition improves mitochondrial function. H9c2 cells were pre‐treated with/without ABT‐702 and exposed to H/R injury. A and B, ΔΨm was detected by JC‐1 staining. (n = 5). Scale bar = 20 μm. C and D, Opening of mPTP was determined by calcein‐AM staining. (n = 5). Scale bar = 20 μm. E and F, Mitochondrial superoxide production was determined by MitoSox staining (n = 5). Scale bar = 100 μm. G, ATP levels were tested by ATP Assay Kit. (n = 5). H, Morphology of mitochondria was observed with transmission electron microscopy. (n = 5). Scale bar = 0.5 μm

Because mitochondria are key regulators of myocardial I/R injury and CaMKII promotes mPTP opening, which is an important mitochondrial function regulator, changes in mitochondrial function were investigated to further explore the effects of ADK inhibition.^34^, ^35^ H/R injury induced depolarization of the mitochondrial membrane as indicated by ΔΨm, whereas ADK inhibition notably improved it in H9c2 cells (Figure 6A,B). As the decline in ΔΨm is due to the opening of the mPTP, Calcein‐AM was used to detect mPTP opening. H/R injury promoted the opening of the mPTP, while this change was prevented by ADK inhibition in H9c2 cells (Figure 6C,D). Mitochondrial superoxide production was also reduced by ADK inhibition (Figure 6E,F). Moreover, ADK inhibition increased ATP production in H/R‐injured H9c2 cells (Figure 6G). Normal mitochondria were found in control cells, whereas damaged and swollen mitochondria were observed in H/R‐injured cells (Figure 6H). ADK inhibition alleviated H/R‐induced changes in mitochondria (Figure 6H).

## 4 DISCUSSION

Identification of novel mechanisms to reduce cardiomyocyte death is always an attractive strategy to prevent myocardial ischaemia/reperfusion injury.^36^, ^37^ In the current study, ADK inhibition limited myocardial infarct size and improved cardiac function after I/R injury by preventing programmed cell death; both apoptosis and necroptosis were regulated by XIAP. Furthermore, XIAP was phosphorylated and up‐regulated via the A_2B_ adenosine receptor/Akt pathway after ADK inhibition. This study indicated that ADK might be a potential therapeutic target for reperfusion‐induced injury.

Although great effort has been devoted to improve myocardium protection during myocardial infarction including regulating neovascularization^38^, ^39^ and oxidative stress,^40^ programmed cell death contributes to a large proportion of I/R‐induced injury.^37^ Apoptosis plays an important role in the pathogenesis of myocardial I/R injury, and treatment with polycaspase inhibitors reduces infarct size by 21%‐52% following I/R.^26^ Preventing necroptosis also reduces myocardial infarct size after I/R injury, which indicates a critical role of necroptosis.^22^ In the present study, ADK inhibition effectively limited infarct size and improved cardiac function after I/R injury. More interestingly, both apoptotic and necroptotic cell death were decreased by ADK inhibition in cardiomyocytes. ADK inhibition also prevented the opening of the mPTP. Thus, ADK might also have an effect on mitochondrial‐mediated apoptosis or necrosis, which warrants further investigation.^41^

In the present study, ADK was transiently increased in I/R‐injured hearts lasting for approximately 4 hours. As hypoxia induces HIF‐1‐dependent repression of ADK, it can be speculated that reperfusion but not ischaemia contributes to the increased content of ADK.^9^, ^10^ ADK has the highest affinity for adenosine, and minor changes in ADK activity rapidly lead to major changes in the concentration of both intracellular adenosine and extracellular adenosine.^9^ We also found that ADK inhibition or knockdown increased myocardial adenosine concentration as indicated by elevated SAH levels (Figure S7A,B). In addition to adenosine, ADK exerts attenuating effects on phenylephrine and pressure overload induced hypertrophy by regulating the mTORC1 and ERK MAP kinase signalling pathways.^19^, ^21^

Adenosine is an ancient extracellular signalling molecule that regulates nearly all aspects of tissue function.^9^ Adenosine is physiologically present at low levels in the interstitial fluids of tissues and rapidly increases in response to pathophysiological conditions including hypoxia, ischaemia, trauma and inflammation.^42^ Increased extracellular adenosine activates one or more of the four adenosine receptors, A_1_, A_2A_, A_2B_ and A_3_ on targeted cells, which generate various cellular responses to restore tissue homeostasis.^43^ All four receptors are expressed in cardiomyocytes. Consistent with previous studies, we found that heart tissues express the highest level of the A_1_ receptor, followed by the A_2A_ receptor, and have much lower expression of A_2B_ and A_3_ receptors.^44^ Similar trends in the expression of adenosine receptors were also found in HL‐1 atrial myocytes.^44^ In the present study, both A_1_ and A_2B_ receptors were abundantly expressed in H9c2 cardiomyocytes, but only A_2B_ receptors were increased after I/R injury. Although adenosine receptors mediate various responses, activation of each subtype appears to play a protective role in myocardial I/R injury.^45^ Previous studies suggest that A_1_ and A_3_ receptors mediate the protective effect of ADK inhibition on reducing rat myocardial I/R injury.^46^ In contrast, the present study documented that the A_2B_ receptor is critical to the effects of ADK on myocardial I/R injury. We also tested the potential role of the A1 receptor in the signalling pathway through which ADK inhibition takes place. Blockade of the A_1_ receptor also attenuated the changes in XIAP and phosphorylation of XIAP and Akt induced by ADK inhibition (Figure S6D). Thus, the A_1_ receptor might also be important in the protective effects of ADK inhibition on myocardial I/R injury.

cIAP1, cIAP2 and XIAP are three of the eight subtypes of IAPs found in humans.^47^ They are widely expressed in different tissues and function as regulators of programmed cell death, cell migration and inflammation.^29^, ^30^ They contain the baculoviral IAP repeat (BIR) domain, which is responsible for mediating protein‐protein interactions that allow them to directly bind to caspases to inhibit cell death.^30^ They also contain a ubiquitin‐associated domain for binding to poly‐ubiquitin chains and a really interesting new gene (RING) domain that provides them with E3 ubiquitin ligase activity.^30^, ^47^ In the present study, all three IAPs were found in heart tissues but only XIAP was changed by I/R injury and ADK inhibition indicating that XIAP might play a more important role in regulating heart injury than other IAPs. The stability of XIAP is regulated by post‐translational modifications including phosphorylation at SER87 by Akt, phosphorylation at S430 by TANK‐binding kinase 1 or IKKε and phosphorylation at S40 by cyclin‐dependent kinase 1‐cyclin‐B1.^48^ Phosphorylation at SER87 by Akt reduces XIAP autoubiquitylation and increases its stability.^48^ Thus, we tested the effect of ADK inhibition on Akt activation and found that Akt activation contributes to the increased XIAP after ADK inhibition. XIAP directly binds to caspase‐3, caspase‐7 and caspase‐9 and inhibits their activity.^48^ However, in addition to caspase‐3 and caspase‐9, we found that XIAP deficiency also affects the activation of caspase‐8 but the detailed mechanism remains to be further studied.

Although further studies are warranted to measure to what extent cell necroptosis contributes to myocardial I/R injury, necroptosis in cardiomyocytes has been proposed as an important component of the pathophysiology of myocardial injury.^22^, ^49^ Necroptosis, a caspase‐independent programmed necrosis, was first reported in 2005 by Degterev et al^50^ In contrast to general unprogrammed necrosis, necroptosis is a form of active cell death triggered by specific signalling pathways during which the activation of RIP1 and RIP3 is essential.^49^ However, necroptosis can also be induced by some stimulants independent of RIP1 or its kinase activity, whereas RIP3 always participates in the signalling pathway.^49^ Interestingly, ADK inhibition changed the content and activation of RIP3 but not RIP1, indicating that ADK regulates cell necroptosis in I/R‐injured hearts in a RIP1‐independent manner.

Both MLKL and CaMKII are the executors of necroptosis and can be phosphorylated by RIP3.^22^, ^49^ After activation, MLKL molecules experience oligomerization and translocate from the cytoplasm to membranes, mediating membrane permeabilization.^49^ In addition to MLKL, CaMKII can be regulated by RIP3 and involved in ischaemia‐induced myocardial ROS overproduction, opening of the mPTP and necroptosis.^22^ In the present study, the activation of MLKL and CaMKII was prevented by ADK inhibition in I/R‐injured hearts. Although CaMKII is considered to be critical for RIP3‐dependent necroptosis during myocardial I/R injury, the role of MLKL should not be neglected and warrants further study.

## 5 CONCLUSION

In summary, we demonstrated that ADK inhibition contributes to limiting myocardial I/R injury by preventing cell apoptosis and necroptosis in cardiomyocytes through the adenosine receptor/Akt/XIAP pathway. ADK may be a potential therapeutic target for protecting against myocardial damage.

## CONFLICT OF INTEREST

The authors confirm that there are no conflicts of interest.

## AUTHOR CONTRIBUTION

**Wenjun Wang:** Data curation (lead); Formal analysis (lead); Investigation (lead). **Bailu Wang:** Data curation (supporting); Methodology (supporting); Resources (lead). **Shukun Sun:** Data curation (supporting); Formal analysis (supporting). **Shengchuan Cao:** Methodology (supporting); Software (supporting). **Xiaoxuan Zhai:** Data curation (supporting); Formal analysis (supporting); Methodology (supporting). **Chuanxin Zhang:** Data curation (supporting); Resources (supporting). **Qun Zhang:** Data curation (supporting); Resources (supporting). **Qiuhuan Yuan:** Data curation (supporting); Formal analysis (supporting); Investigation (supporting). **Yi Sun:** Resources (supporting). **Mengyang Xue:** Methodology (lead); Resources (supporting). **Jingjing Ma:** Data curation (supporting); Investigation (lead). **Feng Xu:** Supervision (supporting). **Shujian Wei:** Conceptualization (lead); Supervision (lead); Writing‐original draft (lead); Writing‐review & editing (lead). **Yuguo Chen:** Funding acquisition (lead); Supervision (equal).

## Supporting information

Supplementary Material

## Data Availability

The data that support the findings of this study are available from the corresponding author upon reasonable request.
