# Supplementary material for: Inhibition of adenosine kinase attenuates myocardial ischaemia/reperfusion injury
Source: J Cell Mol Med. 2021 Feb 1;25(6):2931–43. doi: 10.1111/jcmm.16328 (PMC7957171; doi:10.1111/jcmm.16328)
Supplement: Supplementary file 1 — Supplementary Material [file JCMM-25-2931-s001.pdf]

Supplementary data

Supplemental Figures and Figure Legends:

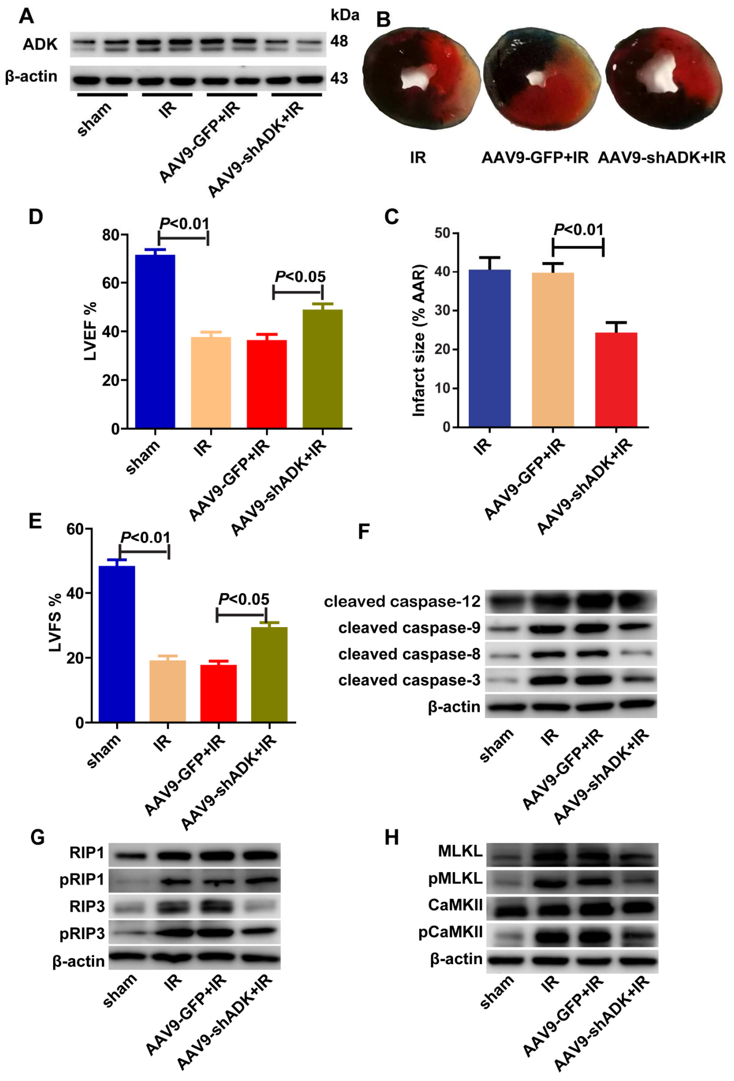

Supplemental Figure 1. Knockdown of ADK alleviates myocardial I/R injury. **A,** AAV9-shADK viruses effectively knocked down the expression of ADK indicated by western blots. **B and C,** Myocardial infarct size were evaluated with Evans Blue/TTC staining and were quantitatively analyzed. (n=8). **D and E,** Cardiac function were evaluated via echocardiography, as indicated by LVEF and LVFS at the end of the 24-hour reperfusion. (n=8). **F-H,** Protein lysates from I/R injured hearts (30 min ischemia/4 h reperfusion) were immunoblotted. (n=5).

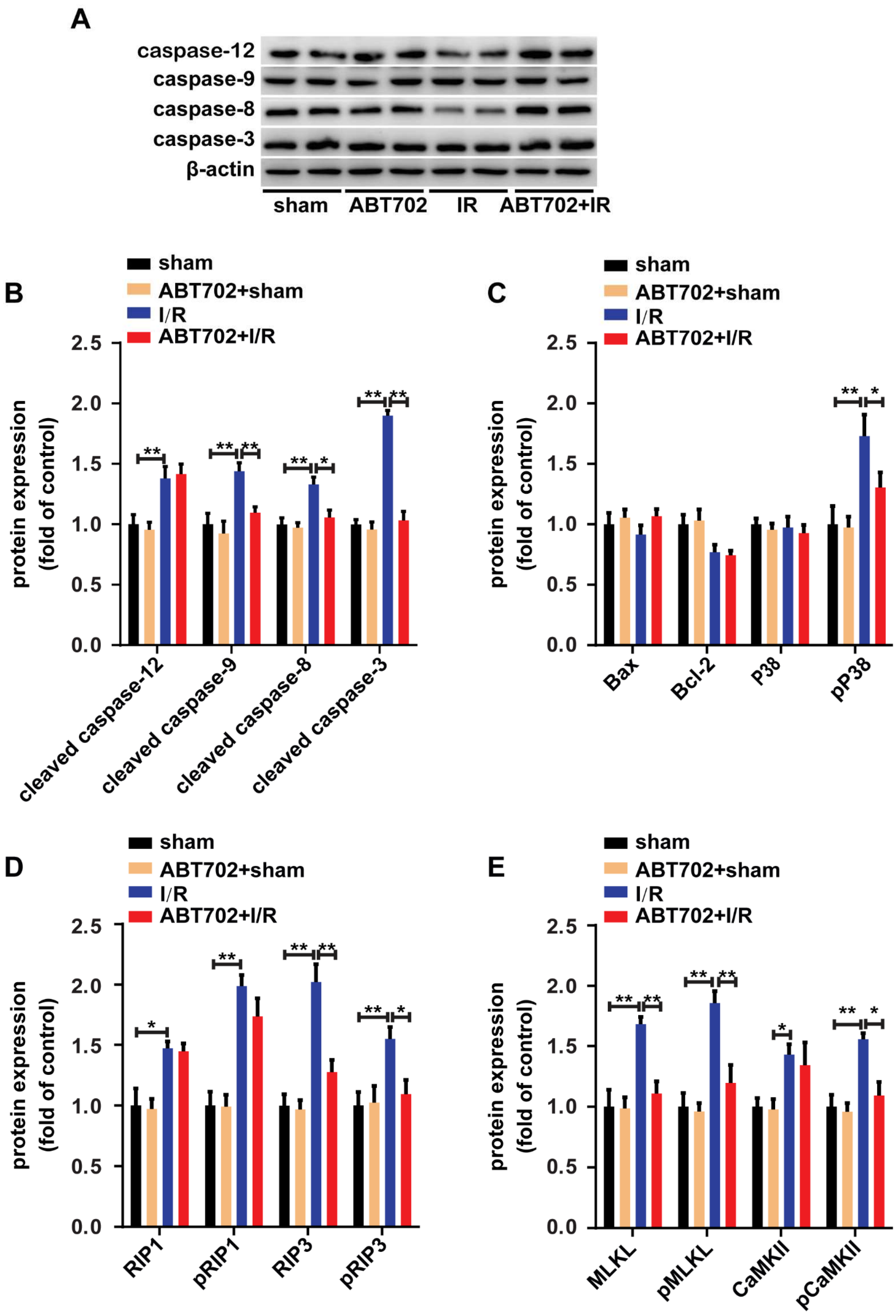

Supplemental Figure 2. A, Representative blots of total protein of caspases in Figure 2C. (n=3). **B,** Quantitative analysis of cleaved caspase-12, cleaved caspase-9, cleaved caspase-8 and cleaved caspase-3 in Figure 2C. **C,** Quantitative analysis of Bax, Bcl-2, P38 and pP38 in Figure 2D. **D,** Quantitative analysis of RIP1, pRIP1, RIP3 and pRIP3 in Figure 2G. **E,** Quantitative analysis of MLKL, pMLKL, CaMKII and pCaMKII in Figure 2H. **P*<0.05, ***P*<0.01 vs control.

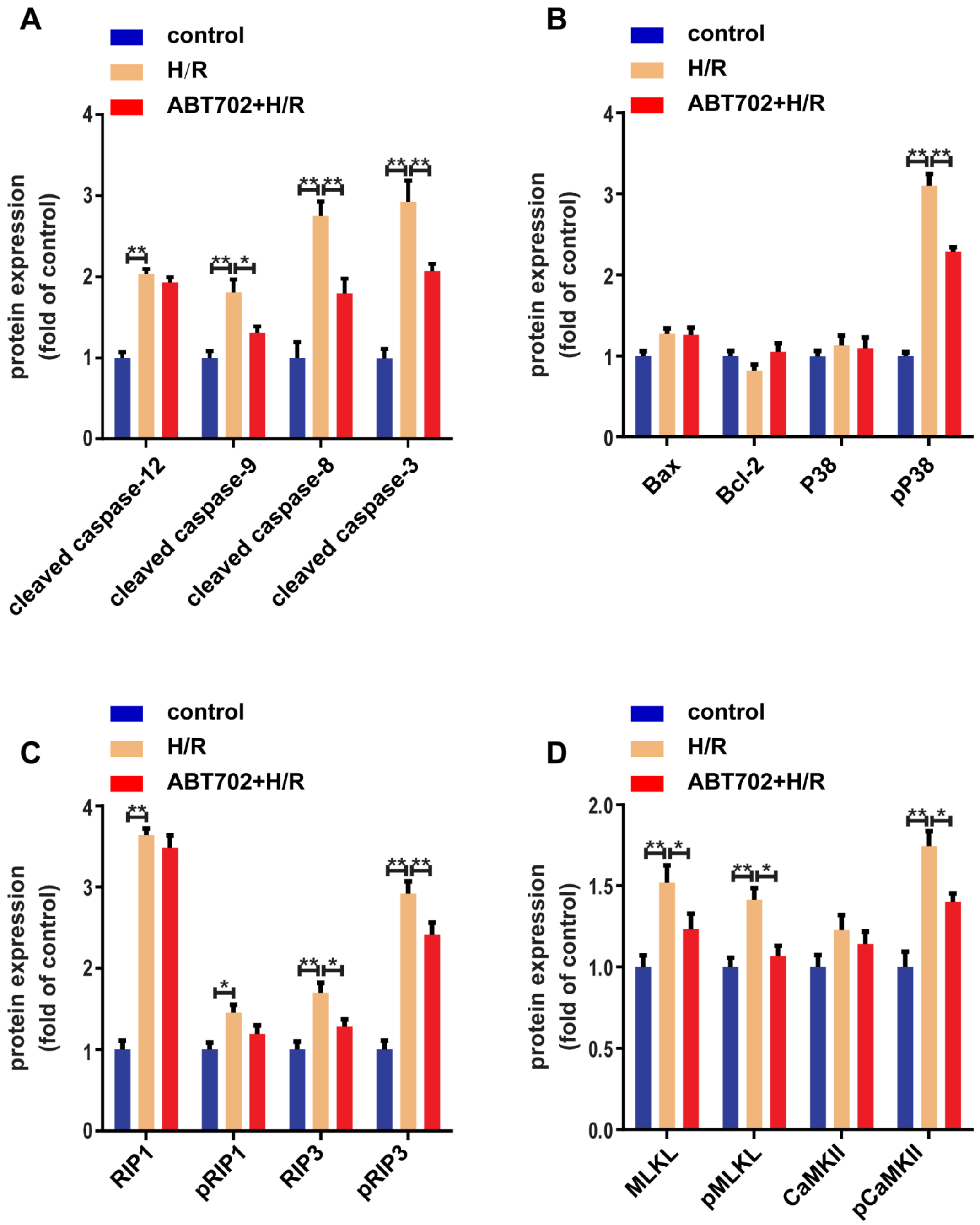

Supplemental Figure 3. A, Quantitative analysis of cleaved caspase-12, cleaved caspase-9, cleaved caspase-8 and cleaved caspase-3 in Figure 3C. **B,** Quantitative analysis of Bax, Bcl-2, P38 and pP38 in Figure 3D. **C,** Quantitative analysis of RIP1, pRIP1, RIP3 and pRIP3 in Figure 3G. **D,** Quantitative analysis of MLKL, pMLKL, CaMKII and pCaMKII in Figure 3H. **P*<0.05, ***P*<0.01 vs control.

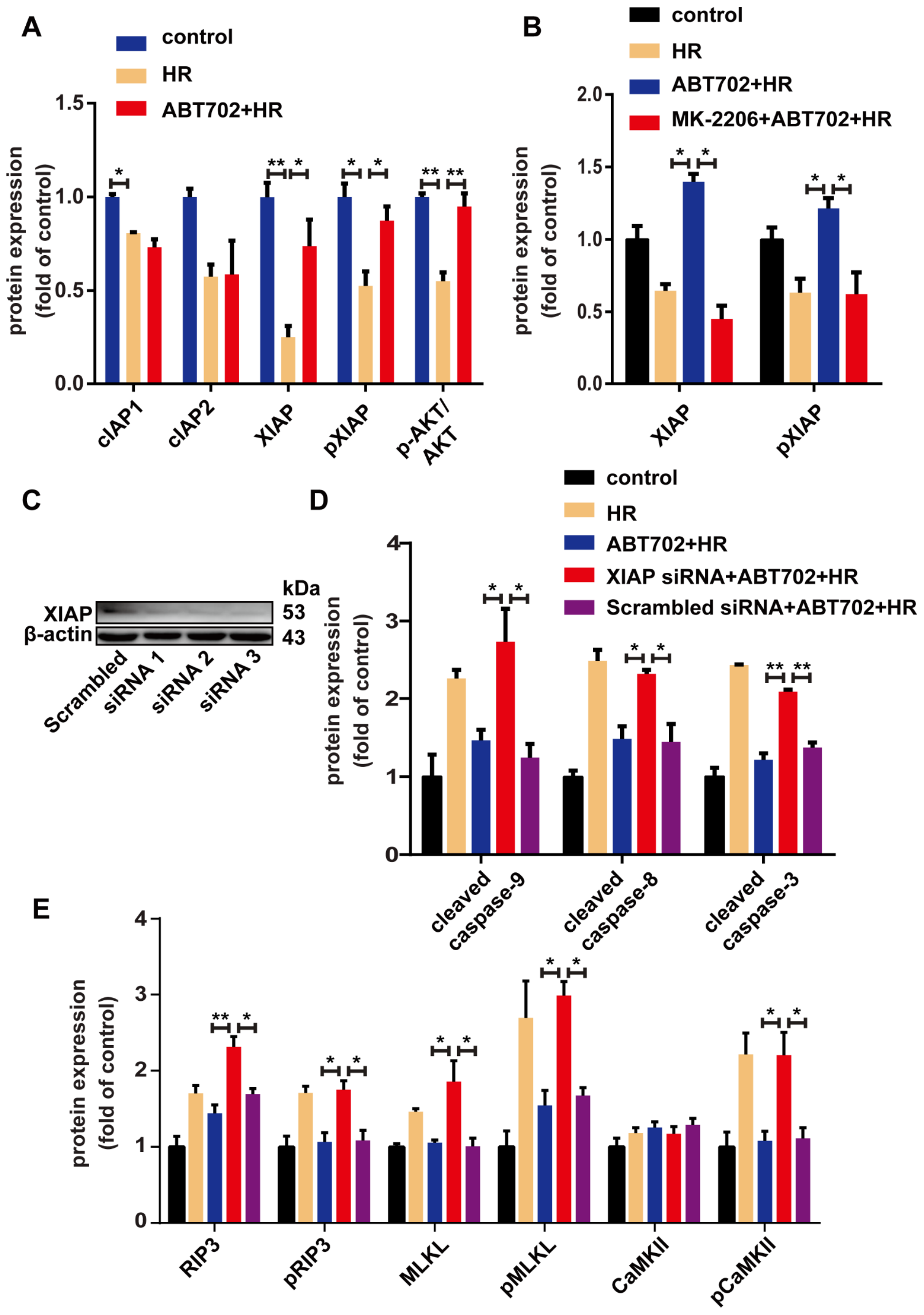

Supplemental Figure 4. A, Quantitative analysis of cIAP1, cIAP2, XIAP, pXIAP and pAKT/AKT in Figure 4A and B. **B,** Quantitative analysis of XIAP and pXIAP in Figure 4C. **C,** Efficiency of XIAP-siRNA were determined by western blotting. (n=3). **D,** Quantitative analysis of cleaved caspase-9, cleaved caspase-8 and cleaved caspase-3 in Figure 4E. **E,** Quantitative analysis of RIP3, pRIP3, MLKL, pMLKL, CaMKII and pCaMKII in Figure 4G. *P<0.05, **P<0.01 vs control.

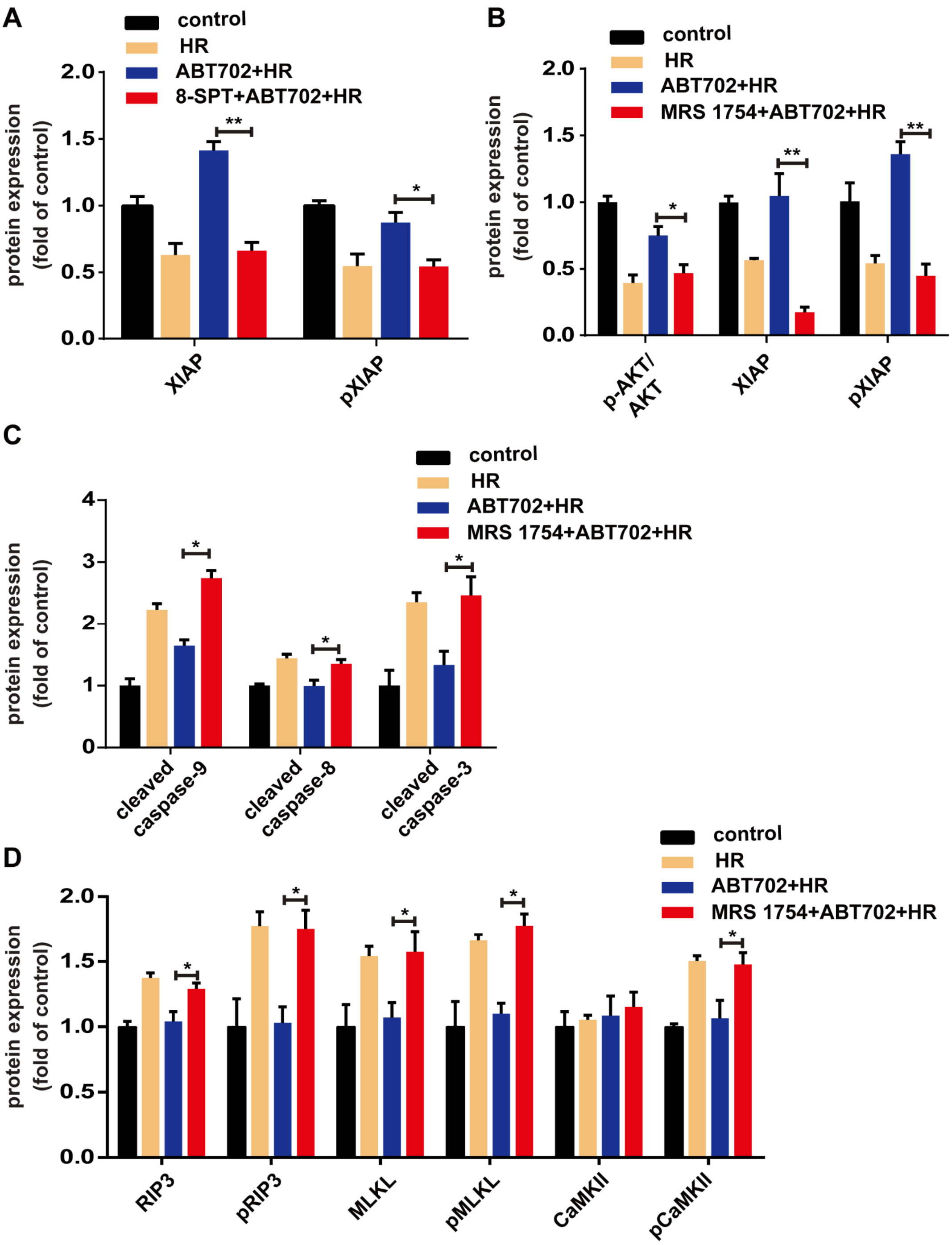

Supplemental Figure 5. A, Quantitative analysis of XIAP and pXIAP in Figure 5A. **B,** Quantitative analysis of pAKT/AKT, XIAP and pXIAP in Figure 5E. **C,** Quantitative analysis of cleaved caspase-9, cleaved caspase-8 and cleaved caspase-3 in Figure 5G. **D,** Quantitative analysis of RIP3, pRIP3, MLKL, pMLKL, CaMKII and pCaMKII in Figure 5H. **P*<0.05, ***P*<0.01 vs control.

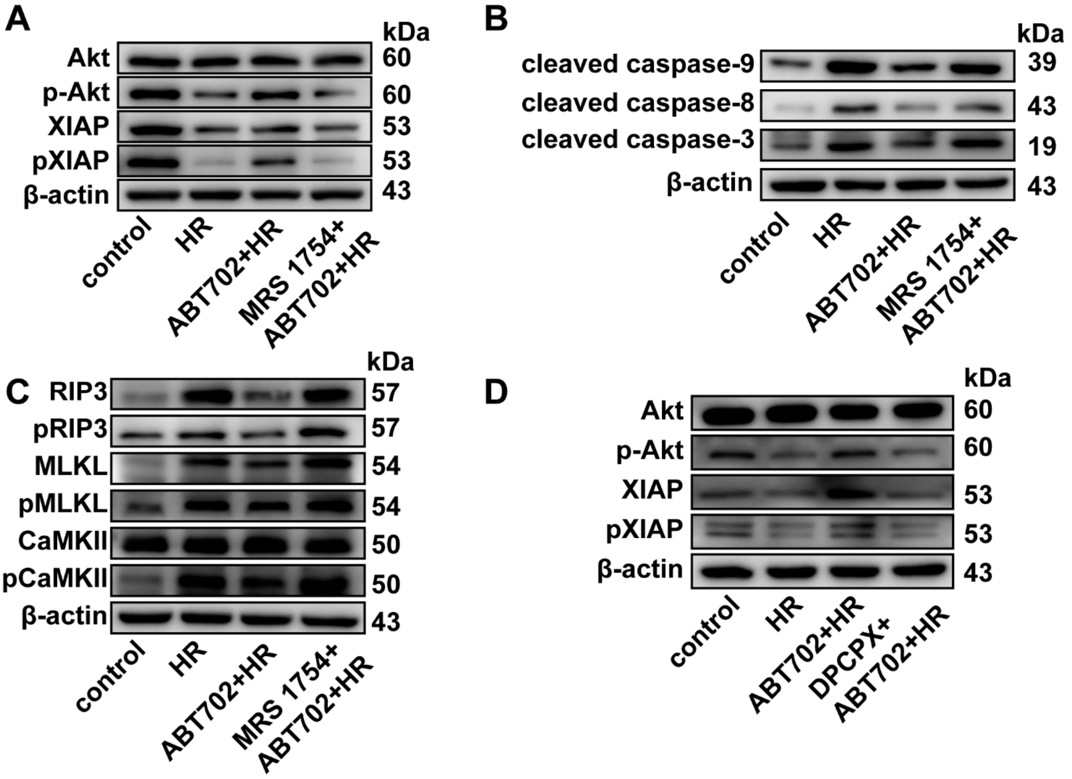

Supplemental Figure 6. A-C, Neonatal rat cardiomyocytes were pretreated with ABT 702 and/or MRS 1754 and then were exposed to 12 h hypoxia and 4h reoxygenation. Lysates were immunoblotted. (n=3). **D,** H9c2 cells were pretreated with ABT-702 and/or DPCPX (10 nM) and were exposed to H/R injury. Lysates were immunoblotted. (n=5).

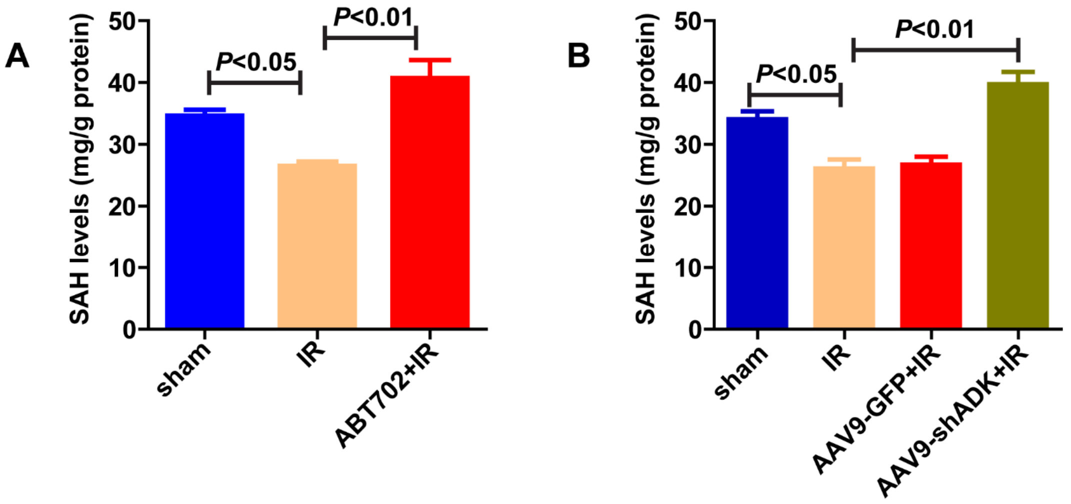

Supplemental Figure 7. A and B, SAH levels were measured in I/R injured mouse heart tissues pretreated with ADK inhibition (2mg/kg ABT702) or ADK knockdown (AAV9-shADK viruses). (n=5).

Supplemental Table 1: XIAP-siRNA sequences

XIAP-siRNA	sequences (5′→3′)
1	GCCAGACUAUGCCCAUUUATT (sense)
	UAAAUGGGCAUAGUCUGGCTT (anti-sense)
2	CGGAGACUCAUGCAGAUUATT (sense)
	UAAUCUGCAUGAGUCUCCGTT (anti-sense)
3	GCACAGUCAUUACGUUCAATT (sense)
	UUGAACGUAAUGACUGUGCTT (anti-sense)

Supplemental Table 2: Primer sequences for adenosine receptors

adenosine receptor A_1 (mice)	forward	5’-ATCTTCCTCACACACGGCAA-3’
	reverse	3’-ACCCGGAACTTGTGGATTCG-5’
adenosine receptor A_1 (rat)	forward	5’-ATCCCTCTCCGGTACAAGAC-3’
	reverse	3’-CACTCAGGTTGTTCCAGCCA-5’
adenosine receptor A_{2A} (mice)	forward	5’-TCATTGCGATTTGCTGGGTG-3’
	reverse	3’-GGGAGCAACACAAAAGCGAA-5’
adenosine receptor A_{2A} (rat)	forward	5’-CCATCCCCTTCGCTATCACC-3’
	reverse	3’-AAGCCATTGTACCGGAGTGG-5’
adenosine receptor A_{2B} (mice)	forward	5’-TCTTTAGCCTCTTGGCGGTG-3’
	reverse	3’-AAACCTTTATACCTGAGCGGGA-5’
adenosine receptor A_{2B} (rat)	forward	5’-ACTCTTCGCCATCCCCTTTG-3’
	reverse	3’-CCTTTATACCTGAGCGGGACG-5’
adenosine receptor A_3 (mice)	forward	5’-GTGCTGCTGATCTTCACCCA-3’
	reverse	3’-GAGTGGTAACCGTTCTATATCTGAC-5’
adenosine receptor A_3 (rat)	forward	5’-TGCATTTTACGGTCGGGAGT-3’
	reverse	3’-TGCAGGCGTAGACAATAGGG-5’
